# Mesmerism in Surgery

**Published:** 1847-01

**Authors:** James Esdaille

**Affiliations:** Civil Assistant Surgeon, H. C. S., Bengal


*Mesmerism in India, and its Practical Applicatious in Surgery and Medicine. By* JAMES ESDAILLE, M. D., *Civil Assistant Surgeon, H. C. S., Bengal*—We have in a preceding article stated the reason why any amount of curative power which mesmerism may possess is not available to the profession; and we trust we shall not be deemed inconsistent in stating a case in which it may be resorted to, Most will acknowledge that, in certain individuals, a more or less prolonged insensibility may be induced by mesmeric procedures, and it is obvious, that the fact of this being so complete as to admit of the performance of surgical operations during its existence, and of surgeons availing themselves of such suspension of sensibility, says nothing *pro* or *con* concerning the remedial agency or the ultra pretensions of mesmerism. The same state has been induced by other means, such as injuries to the head, narcotics and intoxication, during which unfelt operations have been completed. The questions then we have to decide are whether mesmerism can induce such sufficient insensibility, and whether the patient's eventually well-doing is at all compromised by the operation being undertaken under such circumstances. Dr. Esdaille adduces seventy-three instances of painless operations of varying degrees of severity, from the introduction of a seton to amputation of an arm or the tearing of a toe-nail, performed upon subjects who could have no inducement, and scarcely intelligence enough, to enter into any plan of collusion, in the presence of very numerous witnesses. Without agreeing with the author in the general favorable estimate he gives of mesmerism, we may state that we believe the cases we have alluded to are entitled to our belief, and that the subject is one of such vast importance as to call for a searching investigation by the authorities at home. This claim of mesmerism admits of easy testing, and we repeat, if its validity is proved, that none of the various monstrous pretensions of this art will be brought in anywise nearer admission, while a vast boon will have been secured to suffering mankind.—*Medico. Chir. Rev.*

